# Supplementary material for: Combinatorial optimization of gene expression through recombinase-mediated promoter and terminator shuffling in yeast
Source: Nat Commun. 2024 Feb 7;15:1112. doi: 10.1038/s41467-024-44997-7 (PMC10850122; doi:10.1038/s41467-024-44997-7)
Supplement: Supplementary file 1 — Supplementary Information [file 41467_2024_44997_MOESM1_ESM.pdf]

Supplementary Information File

Combinatorial optimization of gene expression through recombinase-mediated promoter and terminator shuffling in yeast

Charlotte Cautereels[1,2], Jolien Smets[1,2], Peter Bircham[1,2], Dries De Ruysscher[3,4], Anna Zimmermann[1,2], Peter De Rijk[5,6], Jan Steensels[1,2], Anton Gorkovskiy[1,2], Joleen Masschelein[3,4] and Kevin J. Verstrepen[1,2*]

[1]VIB Laboratory for Systems Biology, VIB-KU Leuven Center for Microbiology, Leuven, 3001, Belgium

[2]Laboratory of Genetics and Genomics, Center of Microbial and Plant Genetics, Department M2S, KU Leuven, Gaston Geenslaan 1, Leuven, 3001, Belgium

[3]Molecular Biotechnology of Plants and Micro-organisms, Department of Biology, Kasteelpark Arenberg 31, box 2438, KU Leuven, Belgium

[4]Laboratory for Biomolecular Discovery & Engineering, VIB-KU Leuven Center for Microbiology, Leuven, 3001, Belgium

[5]Neuromics Support Facility, VIB Center for Molecular Neurology, VIB, Antwerp, 2610, Belgium

[6]Neuromics Support Facility, Department of Biomedical Sciences, University of Antwerp, Antwerp, 2610, Belgium

[*]Correspondence: kevin.verstrepen@kuleuven.be

This PDF file includes:

Supplemental Figures 1-10

Supplemental Tables 1-4

Supplemental Figures

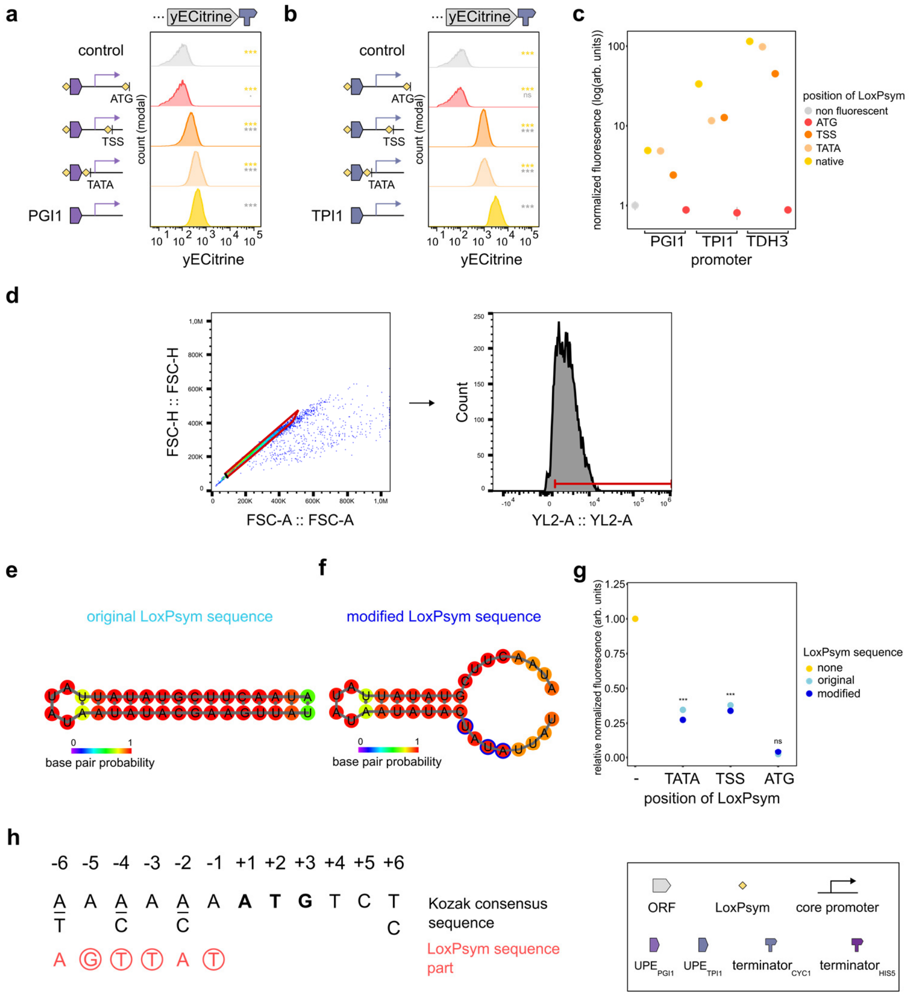

Supplemental Figure 1: Introducing LoxPsym and modified LoxPsym sites at different positions of a yeast promoter reduces protein levels. a. A weak (*PGI1*) and **b.** intermediate yeast promoter (*TPI1*) were used to determine the effect of LoxPsym insertions at various promoter positions on yECitrine fluorescence. LoxPsym (5'-ATAACTTCGTATATTATATAATATACGAAGTTAT-3') was placed at three different locations: directly upstream of the start codon (ATG), the transcription start site (TSS) or the TATA box, as shown by schematic representations on the left. Constructs were tested in combination with terminator *CYC1* and integrated at the *CAN1* locus of *BY4741-mCherry.* The control strain at the top (grey) represents the non-fluorescent strain *BY4741-mCherry.* Statistics by analysis of variance (ANOVA) and two-sided Tukey multiple comparisons of means. Color of the p-values indicate the population which was used for comparison. Full statistical analysis is shown in Supplementary Data 1, *p*-values LoxPsym position. **c.** Comparison of the effect of the LoxPsym site in all three tested yeast promoters on the normalized fluorescence (logarithmic scale). Dots and error bars represent average and standard deviation of three biological replicates. **d.** Two gating steps (red) were performed for during data analysis to obtain single cells (left) and select the mCherry control fluorescence (right, see Methods). **e.** Secondary structure of the LoxPsym RNA sequence with a minimum free energy of -15.40 kcal/mol, as predicted by the RNAFold WebServer, version 2.4.18. Color of the nucleotide indicates

the base pair probability calculated by the algorithm. **f.** Secondary structure of the LoxPsym site with modifications in one of the palindromic repeats, the three modified nucleotides are indicated with a dark blue circle. A previous study showed that this modified LoxPsym arm could still be recombined efficiently by a slightly modified Cre recombinase (with modifications N3D, D29G, L284Q). Structure and the minimum free energy (-4.00 kcal/mol) are determined similarly as in panel **e**. **g.** Comparison of inserting LoxPsym (light blue) and modified LoxPsym (dark blue) at different positions of the promoter sequence (directly upstream of TATA, TSS or ATG). Dots and error bars represent average and standard deviation of three biological replicates. Statistics by ANOVA and two-sided Tukey multiple comparisons of means with *p* = 0.0000001, 0.0000790 and 0.06840, respectively. **h.** Alignment of the Kozak consensus sequence (black, start codon shown in bold) and the 3' end of the LoxPsym sequence (red) when inserted directly in front of ATG. Circles indicate deviations from the consensus sequence. Source data for this figure are provided as a Source Data file.

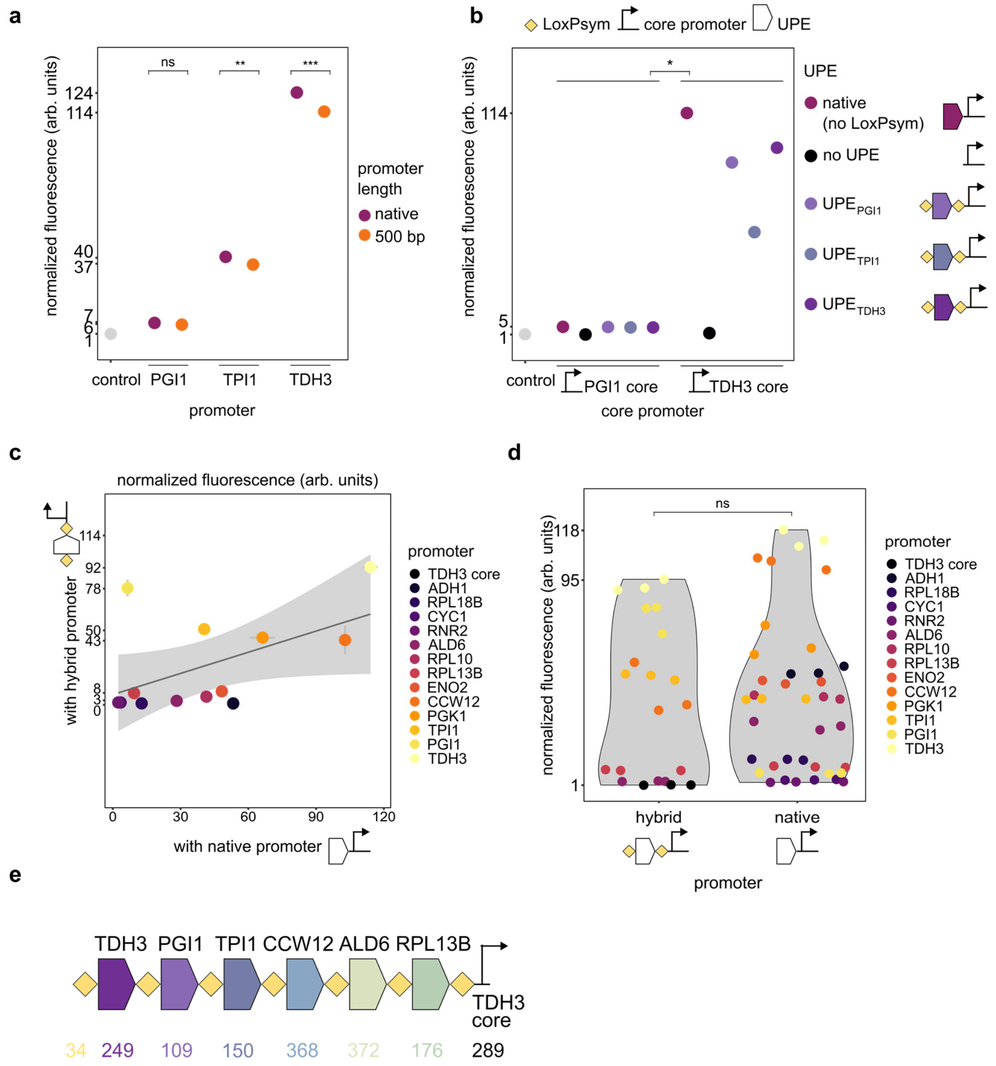

Supplemental Figure 2: Defining UPEs and core promoter for construction of the 5' GEM. a. Effect of promoter length - natural occurrence (purple) or limited to 500 bp (orange) - on the expression of *yECitrine*. Promoter sequences were tested in combination with the *CYC1* terminator and the fluorescent cassettes were integrated at the *CAN1* locus of *BY4741-mCherry*. The control (grey) represents strain *BY4741-mCherry* (no *yECitrine* cassette). Dots and bars represent average and standard deviation of the normalized fluorescence of three biological replicates. Statistics by two-sided Tukey multiple comparisons of means with p = 0.9206, 0.008786 and 0.0000017 for *PGI1*, *TPI1* and *TDH3* promoters, respectively. **b.** Comparison of two core promoters (*PGI1* left and *TDH3* right) combined with their native upstream promoter element (purple), no UPE (black) or the LoxPsym-flanked UPE of *PGI1* (light purple), *TPI1* (blue) or *TDH3* (dark purple). Schematics at the right indicate the layout of the tested constructs, with a variable core promoter (either that of *PGI1* or *TDH3*). Statistics by Fligner-Killeen test with p = 0.02547 to compare the variance of the samples using the *PGI1* core (variance 3.370) or the *TDH3* core (variance 1868). **c.** Correlation between the yECitrine fluorescence measured when expression was controlled by the native *S. cerevisiae* promoter (x-axis) or by the hybrid UPE$_x$-core$_{TDH3}$ promoter (y-axis). Dots and bars represent average and standard deviation of the normalized fluorescence of three biological replicates. Linear regression with $R^2 = 0.29$ and p = 0.05838 (Pearson correlation test). **d.** Violin plots for comparison of the range in expression

caused by hybrid promoters (i.e. LoxPsym flanked UPEs combined with the *TDH3* core promoter, left) or native promoters of *S. cerevisiae* (right). Dots represent the normalized fluorescence of one biological replicate and the color indicates the promoter sequence tested (three biological repeats per promoter sequence). Statistics by Fligner-Killeen test with p = 0.7676 to compare the variance in fluorescence spanned by the building blocks of GEM (variance 1054) or by our selection of native *S. cerevisiae* promoters (variance 1272). **e.** Final layout of the promoter part of GEM, consisting of six LoxPsym (yellow diamonds) flanked UPEs upstream of the *TDH3* core promoter. The numbers below each UPE indicates the length (bp) of the fragment. The sequences of UPEs can be found in Supplementary Data 1, promoter sequences. Source data for this figure are provided as a Source Data file.

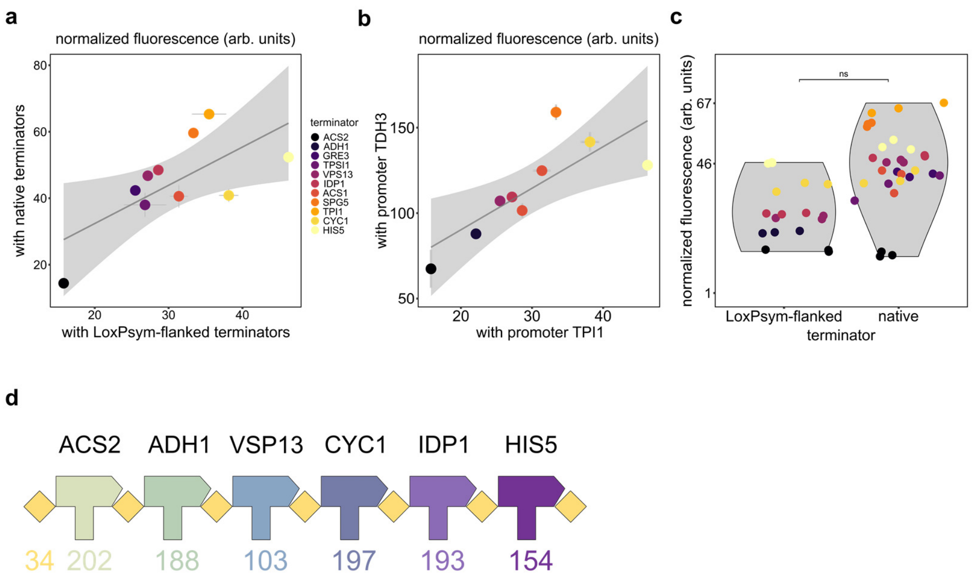

Supplemental Figure 3: Selection of diverse terminator building blocks to include in the design of GEM. a. Correlation between the yECitrine fluorescence measured when expression was controlled by a LoxPsym-flanked terminator (x-axis) or by a natural *S. cerevisiae* terminator (y-axis). Dots and bars represent average and standard deviation of the normalized fluorescence of three biological replicates. Colors of the dots indicate the tested terminator sequence (same legend used in panels **b** and **c** as well). Linear regression with R^2 = 0.47, p =0.02928 (Pearson correlation test). **b.** Correlation between normalized fluorescence obtained under control of the *TPI1* (x-axis) and the *TDH3* (y-axis) promoter in combination with different LoxPsym flanked terminators regulating the expression of *yECitrine*. Linear regression with R^2 = 0.61 and p = 0.01308 (Pearson correlation test). **c.** Violin plots for comparison of the range in expression caused by terminators with (left) or without (right) LoxPsym sites surrounding the terminator. Dots represent the normalized fluorescence of one biological replicate and the color indicates the terminator sequence tested (three biological repeats per terminator sequence). Statistics by Fligner-Killeen test with p = 0.4119 to compare the variance in fluorescence spanned by the 3' GEM-blocks (variance 106.9) or by our selection of *S. cerevisiae* terminators (variance 181.8). **d.** Final layout of the terminator part of GEM, consisting of six LoxPsym (green diamonds) flanked terminators (T). The numbers below each terminator indicate the length (bp) of the fragment. The full sequence can also be found in Supplementary Data 1, terminator sequences. Source data for this figure are provided as a Source Data file.

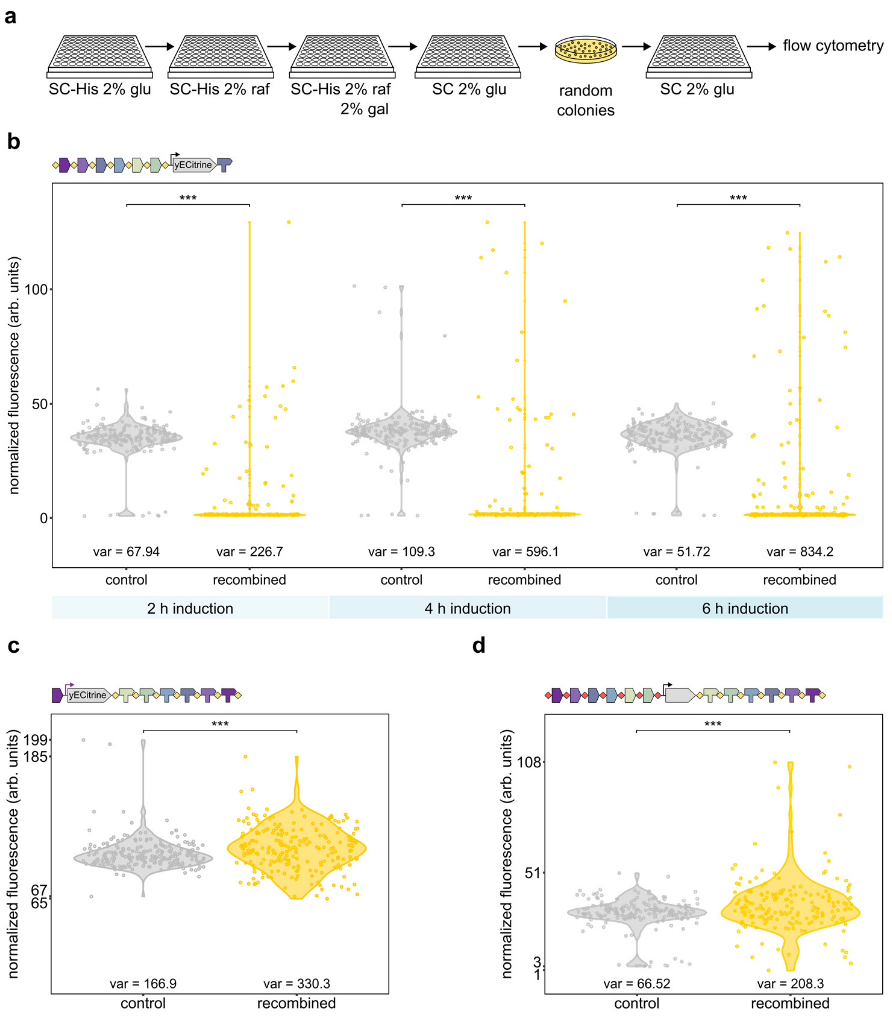

Supplemental Figure 4: Optimization of the induction of GEMbLeR. a. Experimental set-up for induction of GEMbLeR. Cells were consecutively grown for an overnight (ON) in SC-His 2 % glucose (glu) and 2 % raffinose (raf), after which cells were induced in SC-His 2% raffinose 2% galactose (gal). After induction, cells were plated and single colonies were inoculated in SC 2% glu to measure fluorescence with the flow cytometer. **b.** Normalized yECitrine fluorescence of single colonies picked after 2, 4 and 6 h of induction of recombination, by growth in SC 2 % galactose. Strains had the 5’ GEM construct with the UPE array integrated (top) in front of the *yECitrine-tCYC1* construct at the *CAN1* locus and carrier either the pSH47-His-Cre plasmid (expressing *Cre* under control of the *GAL1* promoter, yellow) or the pSH47-His-Vec plasmid (the control plasmid without *Cre*, grey). Six independent populations were induced and plated. Fluorescence of individual clones was measured using flow cytometry. The variance (var) of each induced group is given at the bottom. Statistics by Fligner-Killeen test for variance comparison, with p = 7.20e-07, 7.81e-16 and 4.69e-14 for the induction period of 2, 4 and 6 h, respectively. **c.** Normalized fluorescence of single colonies obtained after 6 h of recombination induction in strains with integration of the *yECitrine* reporter at *CAN1*, under control of promoter *TDH3* and the 3’ GEM construct (top). Statistics by Fligner-Killeen test with p = 3.80e-13. **d.** Normalized fluorescence of single colonies obtained after 6 h of recombination induction in strains with the *yECitrine* reporter at *CAN1*, under control both 5’ and 3’ GEM constructs (top). Note that the LoxPsym sequences differ from those used in the GEM design of **Fig. 3 c**, with sequences 5’-ATAACTTCGTATACACTAGTGTATACGAAGTTAT-3’ (red diamond) and 5’-

ATAACTTCGTATATTATATAATATACGAAGTTAT-3’ (yellow diamond). Statistics by Fligner-Killeen test with p = 8.25e-09. Source data for this figure are provided as a Source Data file.

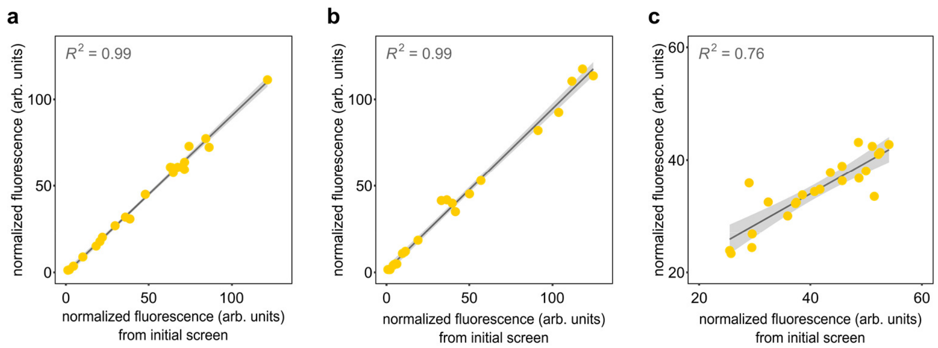

Supplemental Figure 5: Verification of individual, sequenced GEMbLeR variants. Correlation between the initial fluorescence measurements resulting from the large-scale screen of single GEMbLeR variants (x-axis) and the verification after storage of these clones (y-axis). Data shown represent the average normalized fluorescence of 3 biological replicates (y-axis) and the single, initial measurement (x-axis). Vertical bars represent standard deviation. Panels show correlations for the GEMbLeR variants containing a structurally varied **a.** 5' GEM, **b.** 3' GEM and **c.** a combination of both. Statistics by Pearson correlation test with p = 3.0866e-24, p = 7.941e-22 and p = 6.431e-08 for panels **a**, **b** and **c**, respectively. Source data for this figure are provided as a Source Data file.

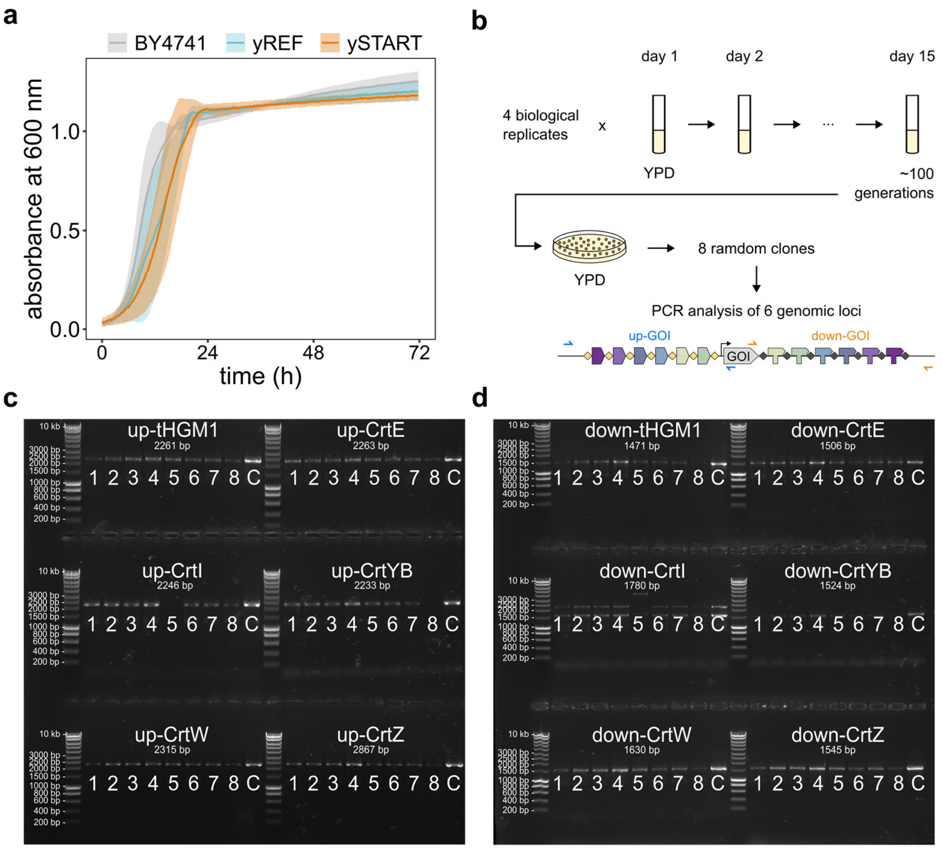

Supplemental Figure 6: Growth profile and evolution of the astaxanthin production strain with heterologous gene expression controlled by GEM. a. Growth profile of the lab strain *BY4741* and derived strains expressing the 6 heterologous genes needed for astaxanthin production in *S. cerevisiae*, either controlled by commonly used promoters and terminators (yREF) or by 5' and 3' GEMs (ySTART). Strains were grown for 72 h in YPD 2 % glucose and absorbance was measured every 15 minutes using the Bioscreen C plate reader. Data indicate the average (lines) and standard deviation (shadows) of four biological repeats per strain. **b.** Experimental setup to verify genomic stability of ySTART after multiple generations. Four independent lineages were inoculated in 5 mL of YPD 2 % and repeatedly diluted to new tubes. OD measurements at 600 nm were used to calculate the number of generations. After the cells reached approximately 100 generations (99.36, 99.31, 99.42, 99.26 for the different lineages), cells were plated and 2 clones per lineage were randomly picked for analysis of the genomic loci where the heterologous pathway genes were integrated. PCR of the up- and downstream GEM (blue and orange primers, respectively) was performed separately to increase resolution by using primers (single arrows) upstream, in and downstream of the GOI. Amplicons 'up-GOI' and 'down-GOI' for the 8 evolved clones (1-8) are shown in panels **c.** and **d.** respectively, together with those of a non-evolved ySTART control strain (C). Amplicon length is indicated for each target and the marker indicates 200 bp – 10 000 bp (SmartLadder MW-1700-10, Eurogentec). Source data for this figure are provided as a Source Data file.

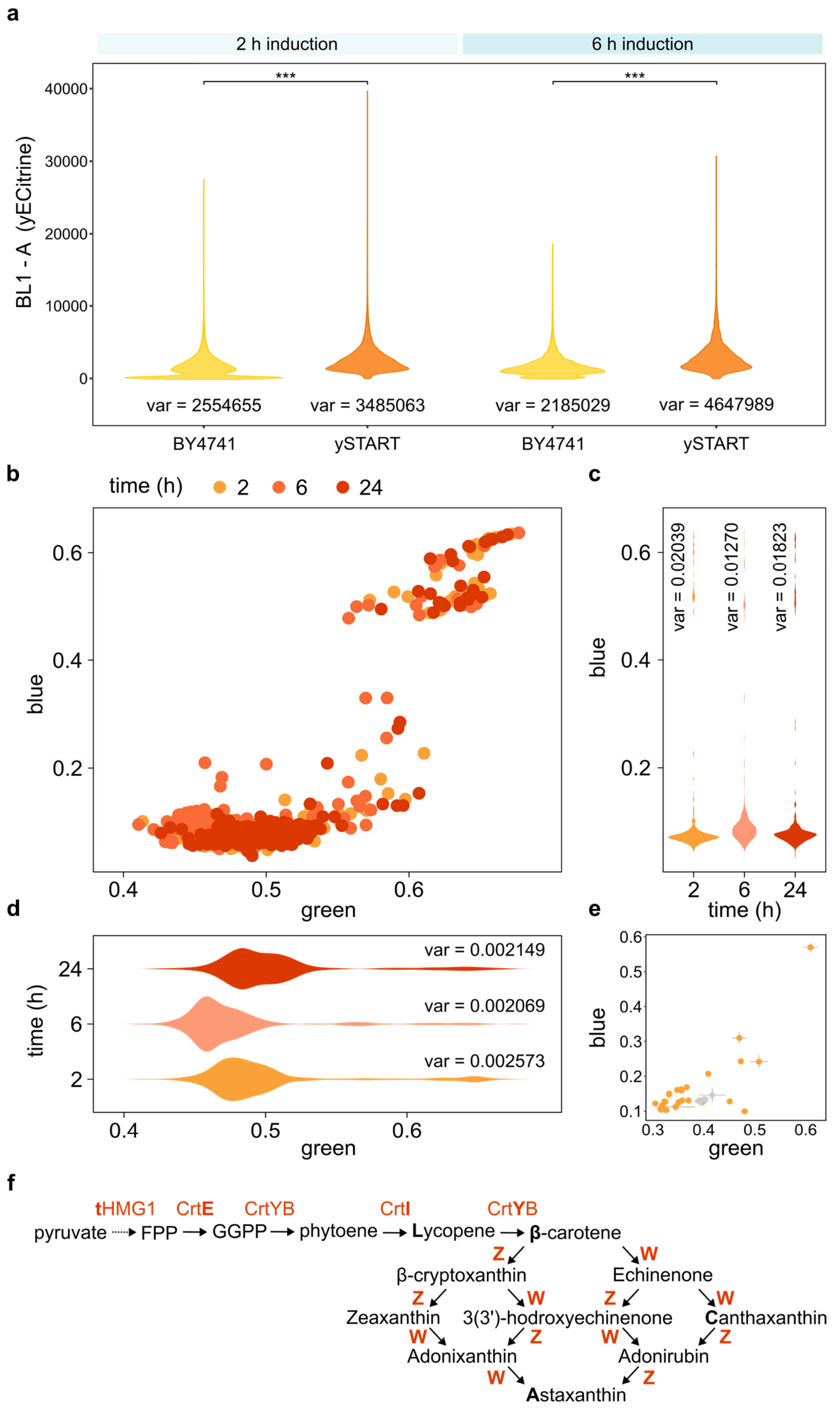

Supplemental Figure 7: Fluorescence and color variation of astaxanthin production strains after different Cre expression induction times. a. yECitrine fluorescence of populations for which expression of the Cre recombinase was induced for 2 h (left) or 6 h (right). yECitrine was expressed in *BY4741* (yellow) or in the astaxanthin production strain ySTART (orange). Data was obtained from three independently induced populations after size gating and concatenated and down-sampled into one group for each sample (N=26354 or 8614) for samples with 2 h and 6 h induction respectively). Statistics by Fligner-Killeen test with p = 2.896e-12 (2 h induction) and 3.705e-10 (6 h induction). **b.** GB-values (blue and green) quantified from randomly picked clones which were plated on YPD after 2

h (yellow), 6 h (grey) and 24 h (blue) of Cre recombinase induction. 307 clones were re-arrayed in 384 well format and grown on SC 2% glucose agar plates at 30 °C for 48 h. Color analysis of plate pictures was used to obtain RGB-values (one biological replicate per data point). Distributions of these values are represented for blue and green in panels **c.** and **d.**, respectively. Variance of each distribution is also depicted (var). Statistics by Fligner-Killeen test was used to determine the difference in variation between induction times with p = 0.000118 (blue) and 0.09412 (green). **e.** GB-values plotted separately for the selection of 30 clones with diversified GEM layout (orange, strain A-D*) and 10 control strains (grey, control 1-10). **f.** Schematic representation of the astaxanthin production pathway, supplementing **Fig. 4 a** to show the colored pathway intermediates formed for the conversion of β-carotene to astaxanthin. Source data for this figure are provided as a Source Data file.

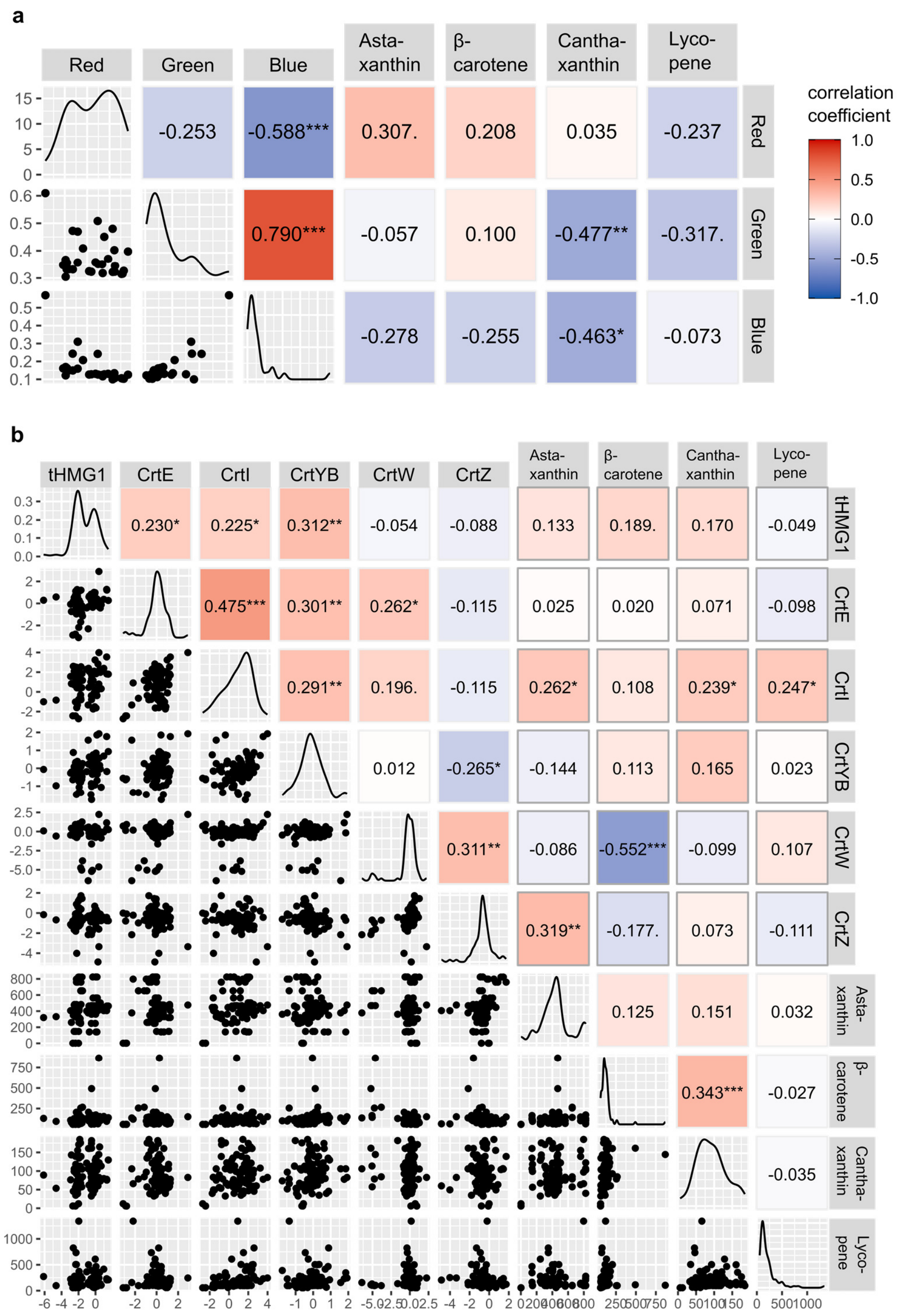

Supplemental Figure 8: Correlation between carotenoid levels, colony colors and astaxanthin biosynthesis gene expression levels from GEMbLeR variants. a. Correlation matrix of RGB-values and carotenoid concentrations (µg/L). Red, Green, Blue values were obtained from image analysis of GEMbLeR variants (biological triplicates measured in technical duplicate). Carotenoid (lycopene, β-carotene, canthaxanthin and astaxanthin) concentrations were measured via LC-MS (three biological repeats per GEMbLeR variant). **b.** Correlation matrix of heterologous gene expression levels (measured by qPCR, three biological repeats per GEMbLeR variant, measured in technical duplicate) and the same carotenoid concentrations (µg/L) used in panel **a**. All statistics by Pearson correlation

test and a list with all p-values can be found in Supplementary Data 1, pairwise correlations. Source data for this figure are provided as a Source Data file.

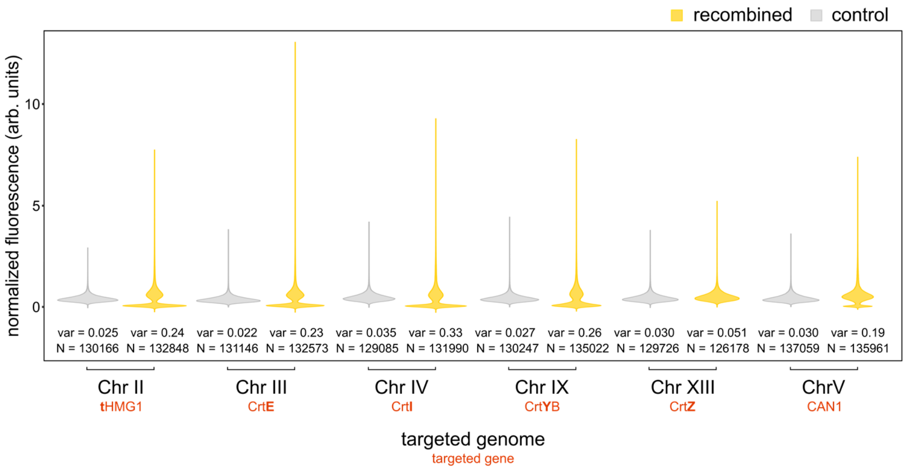

Supplemental Figure 9: GEMbLeR induces expression variation at different genomic loci. Fluorescence is measured at the population level immediately after induction, without plating for single clones. Therefore the normalized fluorescence calculation deviates from those shown above and represents a simple BL1-A/YL2-A (yECitrine/mCherry) division, without normalization for the batch effect. Grey and yellow violins indicate strains with the empty backbone (control) and the *Cre* expression plasmid, respectively. Distributions include the concatenated data obtained from three independently induced biological replicates. Var and N indicate the variance and number of events measured for each group, respectively. The targeted chromosome and specific locus are shown at the bottom and specific integration sites can be found in Supplementary Data 1, genes. Source data for this figure are provided as a Source Data file.

```
# run analysis vs reference refTERMcas
# this is run in genomecomb shell (cg sh)

set expdir ~/exp159254-sacCer3s-Service_Charlotte_Cautereels_BC-
PCR_plate/refTERMcas
cd $expdir
puts "Processing $expdir"

# make reference "genome" from target sequences
mkdir refseq
exec cat {*}[bsort [glob ../sequences-exp159254/*.fa]] | cg fasta2tsv | cg
tsv2fasta > refseq/genome_refseq_refTERMcas.ifas
exec samtools faidx refseq/genome_refseq_refTERMcas.ifas
mklink refseq/genome_refseq_refTERMcas.ifas
refseq/genome_refseq_refTERMcas.fa
exec samtools faidx refseq/genome_refseq_refTERMcas.fa
cg refseq_minimap2 refseq/genome_refseq_refTERMcas.ifas map-ont
exec touch refseq/README.txt

puts "Processing $expdir using the genomecomb pipeline"

set refdir [pwd]/refseq
set logfile $expdir/log_start_[clock format [clock seconds] -format "%Y-%m-
%d-%H:%M"]_[file tail $expdir].txt
if {[catch {
        set ::env(CG_FAST_SHAREDSTORAGE) /buffer/tmp
        exec cg process_project -stack 1 -v 2 -d sge -split 1 \
                -paired 0 -clip 0 \
                -maxfastqdistr 250 \
                -aligner {minimap2} \
                -removeduplicates 0 \
                -realign 0 \
                -distrreg chr \
                -svcallers {sniffles npinv cuteSV} \
                -varcallers {longshot medaka clair3} \
                -methcallers {nanopolish} \
                -sniffles-n -1 \
                -meth_nanopolish-compression gz \
                -threads 6 \
                -reports {fastqstats flagstat_reads samstats histodepth
hsmetrics vars covered histo} \
                $expdir $refdir >& $logfile
} msg]} {
        set o [open $logfile a]
        puts $o "\nError running cg process_project:\n$msg\n$::errorInfo"
        close $o
        file_write $expdir/analysis_startup_error "from run_ont with pid
[pid]\nError starting up $expdir with startup logfile $logfile:
$msg\n$::errorInfo"
        puts "Error starting up $expdir with startup logfile $logfile:
$msg\n$::errorInfo"
} else {
        puts "Started $expdir with startup logfile $logfile"
}

cg long compar/annot_sv-refTERMcas.tsv.zst \
        | cg select -q {$zyg ne ""} -f
{{samplename=regextract($sample,"sample_(\[^_\]+)")}
{method=lindex(split($sample,"-"),0)} chromosome begin end type ref alt zyg
quality DR DV *} \
        > compar/annot_longsv-refTERMcas.tsv
```

```
cg select -q {scount($zyg-clair3-sminimap2 ni "r u" and $quality-clair3-sminimap2 ne "0") > 1 or scount($zyg-longshot-sminimap2 ni "r u") > 1 } -rf {*medaka*} compar/annot_compar-refTERMcas.tsv.zst \
	| cg long \
	| cg select -q {$zyg ne ""} -f {{samplename=regextract($sample,"sample_(\[^_\]+)")} {method=lindex(split($sample,"-"),0)} chromosome begin end type ref alt zyg quality *} \
	> compar/annot_longcompar-refTERMcas.tsv

# depth
# -----
cd ~/exp159254-sacCer3s-Service_Charlotte_Cautereels_BC-PCR_plate/refTERMcas
catch {close $odepth} ; catch {close $oregions}
set odepth [open depth.tsv w]
puts $odepth sample\tchromosome\tposition\tdepth
set oregions [open regions100x.tsv w]
puts $oregions regionsize\tsample
set bams [glob samples/*/map-s*.bam]
set len [llength $bams]
set num 0
foreach bam $bams {
	set sample [file root [file tail $bam]]
	regsub ^map-sminimap2-sample_ $sample {} sample
	regsub _v5.1.15.* $sample {} sample
	puts "[incr num]/$len $sample"
	file_write temp [join {chromosome pos coverage} \t]\n
	# -J Include reads with deletions in depth computation
	# -a -a, -aa Output absolutely all positions, including unused reference sequences
	exec samtools depth -aa -J $bam >> temp
	set regsize [lindex [exec cat temp | cg select -q {$coverage > 100} -g all] end]
	puts $oregions $regsize\t$sample
	set f [open temp]
	gets $f
	while {[gets $f line] != -1} {
		foreach {chromosome pos coverage} [split $line \t] break
		puts $odepth $sample\t$chromosome\t$pos\t$coverage
	}
}
close $odepth
close $oregions

cg select -g {sample * chromosome *} -gc {min(depth),q1(depth),median(depth)} depth.tsv \
    | cg select  -s {chromosome median_depth} > summary_depth.tsv

# nr of reads per chromosome
# --------------------------
cd ~/exp159254-sacCer3s-Service_Charlotte_Cautereels_BC-PCR_plate/refTERMcas
catch {close $o}
set o [open readcnt.tsv w]
puts $o sample\tchromosome\treadcnt
set bams [glob samples/*/map-s*.bam]
set len [llength $bams]
set num 0
foreach bam $bams {
```

```
        set sample [file root [file tail $bam]]
        regsub ^map-sminimap2-sample_ $sample {} sample
        regsub _v5.1.15.* $sample {} sample
        puts "[incr num]/$len $sample"
        set temp [exec cg sam2tsv $bam | cg select -g {chromosome * qname
*} | cg select -g chromosome]
        set temp [lrange [split $temp \n] 1 end]
        list_foreach {chromosome count} $temp {
                puts $o $sample\t$chromosome\t$count
        }
}
close $o
```

Supplemental Figure 10: Code used for Nanopore sequencing data analysis. Guppy basecaller (version 5.1.15) data were analyzed using genomecomb (version 0.103.0), minimap2, samtools, sniffles, npinv, longshot, cuteSV. Sequencing data have been deposited to NCBI Sequence Read Archive database under accession code PRJNA1000506 [https://www.ncbi.nlm.nih.gov/bioproject/?term=PRJNA1000506].

Supplemental Tables

Supplemental Table 1: The environmental condition has a significant effect on the strength of the promoter according to two statistical tests

**function : Pbmodcomp[1]**	
input	fitS=lmer(FLUO~(1|PROM)+(1|MEDIUM),weights=count,data=data)
	fitL=lmer(FLUO~(1|PROM)+(1|MEDIUM)+(1|PROM:MEDIUM),weights=count,data=data)
	PBmodcomp(largeModel = fitL, smallModel = fitS)
output	Requested samples: 1000 Used samples: 961 Extremes: 0<br>LRT = 1240.9, p-value < 2.00e-16 (likelihood ratio test)
**function : exactRLRT**	
input	m=lmer(FLUO~(1|MEDIUM)+(1|PROM:MEDIUM),weights=count,data=data)
	m0=lmer(FLUO~(1|PROM)+(1|MEDIUM),weights=count,data=data)
	mA=lmer(FLUO~(1|PROM)+(1|MEDIUM)+(1|PROM:MEDIUM),weights=count,data=data)
	exactRLRT(m, mA, m0)
output	simulated finite sample distribution of RLRT. (p-value based on 10000 simulated values)<br>RLRT = 1145.8, p-value < 2.2e-16 (restricted likelihood ratio test)

[1] Evaluation of the interaction between the promoter sequence (PROM) and the medium in which the cells were grown (MEDIUM). To asses if the interaction had a significant effect on the observed level of yECitrine fluorescence (FLUO), different linear mixed-effects models (lmer) were built.

Supplemental Table 2: The environmental condition has no significant effect on the strength of the terminator

**function : Pbmodcomp[1]**	
input	fitS=lmer(FLUO~(1|TERM)+(1|MEDIUM),weights=count,data=data)
	fitL=lmer(FLUO~(1|TERM)+(1|MEDIUM)+(1|TERM:MEDIUM),weights=count,data=data)
	PBmodcomp(largeModel = fitL, smallModel = fitS)
output	Requested samples: 1000 Used samples: 480 Extremes: 73<br>LRT = 1.1972, p-value = 0.2739 (likelihood ratio test)
**function : exactRLRT**	
input	m=lmer(FLUO~(1|MEDIUM)+(1|TERM:MEDIUM),weights=count,data=data)
	m0=lmer(FLUO~(1|TERM)+(1|MEDIUM),weights=count,data=data)
	mA=lmer(FLUO~(1|TERM)+(1|MEDIUM)+(1|TERM:MEDIUM),weights=count,data=data)
	exactRLRT(m, mA, m0)
output	simulated finite sample distribution of RLRT. (p-value based on 10000 simulated values)<br>RLRT = 1.0887, p-value = 0.1238 (restricted likelihood ratio test)

[1] Evaluation of the interaction between the terminator sequence (TERM) and the medium in which the cells were grown (MEDIUM). To asses if the interaction had a significant effect on the observed level of yECitrine fluorescence (FLUO), different linear mixed-effects models (lmer) were built.

Supplemental Table 3: Cloning scheme for Gibson assembly of the GEM constructs

**construction of 5' GEM[1]**										
**DNA part**	UPE-*TDH3*	UPE-*PGI1*	UPE-*TPI1*	UPE-*CCW12*	UPE-*ALD6*	UPE-*RPL13B*	core-*TDH3*	*yECitrine*	term-*CYC1*	backbone
**amplified using oligo's[2]**	OF/R1	OF/R2	OF/R3	OF/R4	OF/R5	OF/R6	OF/R7	OF/R8	OF/R9	OF/R10
**construction of 3' GEM[1]**										
**DNA part**	prom-*TDH3* or prom-*TPI1*	*yECitrine*	term-*ACS2*	term-*ADH1*	term-*VSP13*	term-*CYC1*	term-*IDP1*	term-*HIS5*	backbone	
**amplified using oligo's[2]**	OF/R11 or OF/R12	OF/R20	OF/R13	OF/R14	OF/R15	OF/R16	OF/R17	OF/R18	OF/R19	

[1] Sequences of all DNA parts used for cloning can be found in Supplementary Data 1, promoter sequences, terminator sequences and constructs.

[2] Sequences of the primers used for amplification can be found in Supplementary Data 1, oligo's.

Supplemental Table 4 : List of the plasmids used in this study

plasmid nb	plasmid name	expression	vector template	F primer vector	R primer vector	insert template	F primer insert	R primer insert	additional information
P1	pV1382	*S. cerevisiae*	AddGene #111436	–	–	-	–	–	expression of *Cas9* and sgRNA (inserted via cloning)
P2	pSH47-His-Vec	*S. cerevisiae*	Genbank #AF298782	GCGGTGTGAAATACCGCACAGA	CCTGACGGGCTTGTCTGCTC	pSH47-His-Cre	GCGTGACATAACTAATTACATGACTCGAGGGAGCTCCAGCTTTTGTTCCCTTTAGTG	CACTAAAGGGAACAAAAGCTGGAGCTCCCTCGAGTCATGTAATTAGTTATGTCACGC	removal of *Cre* ORF to obtain control plasmid
P3	pSH47-His-Cre	*S. cerevisiae*	Genbank #AF298782	GCGGTGTGAAATACCGCACAGA	CCTGACGGGCTTGTCTGCTC	AddGene #49455	CTCTGACACATGCAGCTCCCG	AGGGATTTTGCCGATTTCGGCC	marker swap on plasmid expressing *Cre* recombinase
P4	pLM494	*S. cerevisiae*	Addgene #100539	–	–	-	–	–	expression of *tHMG1*, *CrtE*, *CrtI* and *CrtYB*
