# Supplementary material for: Combinatorial optimization of gene expression through recombinase-mediated promoter and terminator shuffling in yeast
Source: Nat Commun. 2024 Feb 7;15:1112. doi: 10.1038/s41467-024-44997-7 (PMC10850122; doi:10.1038/s41467-024-44997-7)
Supplement: Supplementary file 3 — Description of Additional Supplementary Files [file 41467_2024_44997_MOESM3_ESM.pdf]

Title: Supplementary Data 1

Description: promoter sequences, terminator sequences, p-values LoxPsym position, genes, pairwise correlations, carotenoids, stats carotenoids, expression, stats expression, strains, oligo’s, constructs, LoxPsym, nanopore
